# Future Directions of Intraoperative Radiation Therapy: A Brief Review

**DOI:** 10.3389/fonc.2017.00300

**Published:** 2017-12-12

**Authors:** Tatjana Paunesku, Gayle E. Woloschak

**Affiliations:** ^1^Department of Radiation Oncology, Feinberg School of Medicine, Northwestern University, Chicago, IL, United States

**Keywords:** intraoperative radiation therapy, immunotherapy, nanotechnology, radiosensitizers, normal tissue injury

## Abstract

The use of intraoperative radiation therapy (IORT) is increasing with the development of new devices for patient treatment that allow irradiation without the need to move the patient from the surgical table. At the moment, ionizing radiation in the course of IORT is supported most often by the use of mobile devices that produce electrons, kilo voltage X-rays, and electronic brachytherapy and the development of applicators suitable for delivery of radionuclides for short-term brachytherapy. The establishment of new treatment devices and protocols that can be foreseen in the future, e.g., the development of proton or heavy ion sources suitable for IORT or the establishment of new treatment protocols such as the use of IORT in combination with immune system modulators or radiosensitizing nanoparticles, could lead to a significant increase in the use of IORT in the future. This review discusses the still limited use of IORT at this point in time and hypothesizes about possible future approaches to radiotherapy.

## Intraoperative Radiation Therapy (IORT) Today

By definition, IORT is delivered to the tumor and tumor bed (preferably to tissues at the 10 mm depth) as they are exposed during surgery to the patient; often, this also includes surgical distancing of normal tissues that could be injured by radiation (e.g., retraction of skin away from the IORT source). Regardless of the dose of radiation delivered, the critical feature of IORT is its precision and minimal exposure to the surrounding healthy tissues. In this manner, IORT fulfills one of the key promises of targeted radiation therapy—it accomplishes minimization of systemic side effects by limiting the irradiated normal tissue volume and increasing the therapeutic index. Thus, IORT has found its use in those situations where surgical intervention provides an opportunity to increase radiation dose to the tumor (dose escalation) and/or decrease total dose to normal tissues (dose de-escalation).

The use of radiation in combination with surgery has a long history. Before the 1960s, the preferred approach to combine these treatment modalities was to do a presurgical radiation treatment. For example, the delivery of 35–40 Gy to the chest wall followed at 6 weeks by mastectomy was a frequent approach for breast cancer patient care at MD Anderson before 1960s (1). Technical difficulties of delivering radiation at the time of surgery were probably the primary reasons why such treatment was attempted for the first time only after 1960. The first IORT work similar to current practice was done in Japan at Kyoto University (2–4); it was soon followed by similar work around the world (5). The successful results of this early work generated much excitement; the first group of IORT gastric cancer patients included two examples of posttreatment 5-year survival of patients with only partially resected gastric cancer who received a 40 Gy intraoperative dose (4). Further developments in the field followed, rapidly at first, with surgical suites built in radiation rooms in some cases (5), and much instrument manipulation done collaboratively between hospitals and manufacturers of IR devices (6). IORT quickly became adopted worldwide despite the technical challenges (2, 7–10) but became less used once IR equipment manufacturers ceased engaging in customized instrument changes. A recent resurgence of this treatment modality can probably be credited to the development of new types of IORT dedicated radiation devices such as mobile linear accelerators (11) and specially designed applicators for positioning of high activity radioactive sources (12). Thus, although current medical device regulations prevent in-house development of IORT accelerators—a problem that slowed this field until recently, the availability of commercial devices obviated this necessity.

Early translational studies, conducted by Abe before the first patient IORT and subsequently by many who worked in this field, found a relatively high resilience of different tissues to IORT treatment. For example, experimental studies in a canine model established IORT doses for abdominal surgeries incompatible with different types of postsurgical healing (13). In a study that included nearly 70 animals, it was determined that the intact large blood vessels tolerate doses up to 50 Gy, the urethra up to 30 Gy, and bile ducts up to 20 Gy. Postsurgery the same tissues became less able to cope with radiation injury. Intestinal sutures and arterial anastomoses were found to heal after doses of 45 Gy although not without occasional fibrotic complications. These and subsequent studies in animal models and data from human patients led to the general conclusion that IORT doses as high as 25 Gy could be tolerated by the majority of normal (not surgically treated) tissues without significant toxicity (14, 15). Among the late effects of IORT, delayed progressive ischemia was found to be the complication of most concern. Most of these radiobiological studies were followed-up with patient work that helped drive the IORT field in general.

While some of the illustrative examples of IORT are discussed here, this document is not intended to provide a thorough overview of IORT in current practice. Several exhaustive reviews of IORT were published in recent years demonstrating obstacles and successes of this field (5, 12, 16, 17), and the diverse group of treatment approaches and cancer types treated by IORT in the past few decades. Conclusions from those studies were positive in all cases—if used in appropriate patient groups and controlled correctly, IORT is a life saving procedure. Patient selection criteria, according to these sources, include the following:

- Low local control rate achievable with surgery alone;
- No medical contraindications for gross tumor resection;
- “Standard” radiotherapy doses required for adequate local control exceeding normal tissue tolerance;
- No evidence of distant metastases.

The last of these criteria (no evidence of distant metastases) is the basis for most successful IORT treatments. Immediate, single dose irradiation after surgery, in low-risk patients who are most likely to be free of metastasis further assures long-term disease free survival of such patients. For example, an IORT study with women stratified into low-risk and high-risk cohorts as defined by the ELIOT trial, found a greater survival and greatest benefits from IORT in the low-risk group (18). While it may be disappointing that IORT is not of greater benefit for high-risk patients (in whom it does not seem to be of much effect), the fact that this treatment improves disease free survival in low-risk patients is one of the most significant arguments in favor of this therapy.

Nevertheless, it should also be recognized that IORT has found its place in other cancer related scenarios. These include supplemental treatment to recurrent cancers (not eligible for external beam radiotherapy), as well as in palliative care. For example, IORT may be used in combination with kyphoplasty in patients with vertebral metastases; few studies using IORT for metastatic disease documented good pain control (16).

A recent review by Pilar and others (12) provides an excellent resource for review of surgical situations known to employ IORT and lists the possible outcomes achieved by IORT under these different circumstances. In this, most recent review of IORT literature, cancer types treated by IORT include following: primary and recurrent head and neck cancers (IORT doses between 10 and 22 Gy, 2-year overall survival 20–60%), breast cancers (wide spectra of cancer types and outcomes), locally advanced colorectal cancers (IORT doses between 10 and 20 Gy, 5-year overall survival up to 75%), soft tissue sarcomas (IORT doses between 7.5 and 30 Gy, 5-year overall survival up to 7–40%), pediatric tumors (mostly neuroblastoma) (IORT doses between 7.5 and 20 Gy, 10-year overall survival up to 74%), gynecological tumors (IORT doses between 8 and 30 Gy, 5-year overall survival up to 47%), bladder and renal cancers (IORT doses between 9 and 20 Gy, 5-year overall survival up to 73%), prostate cancers (IORT doses between 10 and 30 Gy, 5-year overall survival up to 100%), gastric cancers (IORT doses between 12 and 35 Gy, 2-year overall survival up to 47%), and pancreatic cancers (IORT doses between 10 and 33 Gy, 5-year overall survival up to 35%).

## Future Directions in IORT

First, it should be mentioned that the potential combined use of IORT and immunotherapy was recognized in a recent review by Herskind and others (19). Increasing evidence suggests that high single doses of ionizing radiation may result in tumor-directed immune reactions, locally and systemically (20). While no conclusive data about immune reactions and IORT are available, high single doses delivered during IORT may be expected to cause immunogenic cell death (21) of tumor cells remaining *in situ* after surgery. The greatest concern lies with the fact that the numbers of tumor cells remaining after surgery may be too low to trigger a favorable immune system reaction. (In the same vein—it could perhaps be interesting to investigate the data from IORT cases with incomplete surgical resection, such as, for example, unexpected survivors from Abe’s initial studies.) The possibility that IORT may lead to immunogenic cell death of tumor cells may provide an additional reason to investigate IORT anew, perhaps in combination with immune checkpoint blockade treatments (19).

Next, we would like to consider the fact that IORT could easily be combined with other therapies that are also more potent with local delivery and could, in turn, increase radiation sensitivity. IORT is inherently a treatment approach designated for local cancer control. Local delivery is achieved “mechanically”—by placing the source of radiation close to tumor or tumor bed. New therapeutic modalities, still at preclinical testing such as nanoparticle therapies, could also benefit from using a postsurgery scenario to secure targeted local delivery. IORT would then be complemented with localized delivery of nanoparticles that could be designed, e.g., for a controlled release of cargo. Many nanomaterials have the capacity to respond differentially to different temperatures, pH conditions and more; for an extensive review of porous nanomaterials fine tuned for “gated” cargo delivery see, for example Ref. (22). While it may be difficult to guarantee that a given nanoparticle type may not encounter a “cargo-release signal” if delivered systemically, it is much easier to be sure that cargo delivery will be controlled if nanomaterial is to be delivered locally, to tumor or tumor bed physically revealed by the surgery.

It is also not a difficult next step to imagine a potential synergy between radiosensitizing nanoparticles and IORT. In its simplest form—one can envision combined use of IORT with nanoparticles with radiosensitizing small molecule cargo, increasing the efficiency of radiation therapy additively or synergistically. A more complex, and possibly more exciting situation could be accomplished by using nanoparticles with inherent and novel radiosensitizing capacity such as ability to intensify radiation effects by localized release of electrons or reactive oxygen species (23). An overview of different possible radiosensitizing nanoparticle materials (24) details how many Auger electrons, Compton electrons, and photoelectrons are expected for nanoparticles made of elements of different Z and lists the anticipated total energy absorbed and released by each material upon exposure to keV and MV ionizing radiation. Relative biological effectiveness calculated based on these data suggests that keV radiation may be more effective than MV radiation in the presence of nanomaterials rich in Auger electrons. Similarly, several predictions about radiosensitization that can be expected by combining gold nanoparticles with brachytherapy radionuclides (I-125, Pd-103, and Yb-169) and low-energy (50 keV) X-rays were also recently published (25, 26); as well as investigation of potential effects of combination of high Z nanomaterials and proton therapy (27). To all these considerations, one may also add that nanoparticle uptake by cells leads to radiosensitization by biological, cellular, and molecular mechanisms as well (23).

A significant concern in IORT is the dosimetry, and a 10 mm depth margin surrounding the tumor cavity is considered as tissue that must be treated. The current “standard” in IORT linacs that produce MeV electrons are devices called the Mobetron, the Liac, and the Novac; while IORT low-energy X-ray irradiators most frequently used at present are the Intrabeam System and the Papillon System (6). The linacs used for IORT produce electron beams with energies between 4 and 12 MeV with 2–3 MeV steps increase and corresponding penetration increase of 7–10 mm per step; use of higher energies is avoided to prevent neutron contamination that would require additional shielding (6). IORT low-energy X-ray irradiators produce 30 and/or 50 keV X-ray photons and the dose delivered with these devices decreases rapidly with distance; and a prescription at 10 mm depth is considered as best to ensure that desired dose arrives at this tissue depth for all applicator sizes (28). Based on discussion about high Z nanoparticles above, it is clear that they could significantly increase effect of IORT exposures and do so locally—which is the exact prerequisite for IORT.

Finally, it should be mentioned that it might be possible that IORT devices that produce protons or even heavy ions may become possible in the future. These radiation modalities could extend the postsurgical radiation treatment deeper and further than 10 mm from resection margin. While treatment distances further than 10 mm were not considered in IORT practice so far, it is possible that such devices would find their use in clinical practice as well.

## Concluding Thoughts

Intraoperative radiation therapy was originally an innovative and clever approach for delivery of radiation during the course of surgery to reduce normal tissue toxicity and at the same time improve tumor treatment. When the modality was first established, limitations revolved predominantly around the availability of instrumentation, the design of facilities and other physical concerns. Radiobiology done to understand constraints of the technology was limited and focused on available systems. With the deeper understanding of the importance of tumor immunology and the development of new tools in nanotechnology, it is hopeful that IORT will be rediscovered. Additional radiobiology experiments designed to probe possible new uses of IORT are essential to understand potential benefits and limitations of these treatments.

## Author Contributions

TP and GW selected the references and cowrote the text.

## Conflict of Interest Statement

The authors declare that the research was conducted in the absence of any commercial or financial relationships that could be construed as a potential conflict of interest.
